# Occurrence of congenital cerebral theileriosis in a newborn twin Holstein calves in Iran: Case report

**Published:** 2014

**Authors:** Mohammad Tolouei Kaleibar, Javad Ashrafi Helan, Ezzatollah Fathi

**Affiliations:** 1 *Department of Clinical Sciences, Faculty of Veterinary Medicine, University of Tabriz, Tabriz, Iran; *; 2 *Department of Pathobiology, Faculty of Veterinary Medicine, University of Tabriz, Tabriz, Iran.*

**Keywords:** Calves, Cerebral theileriosis, Twin

## Abstract

An 8-day-old newborn female twin Holstein calves with a history of weakness, anorexia, emaciation and convulsion were presented to Tabriz University Veterinary Teaching Hospital. On admission, the calves were febrile and recumbent. Physical examination revealed many ticks from the external body surface of the animals, right and left prescapular lymphadenopathy, severe opisthotonos, nystagmus, pedaling, blindness, hyperpnea and hypersthenia. Buccal and vaginal mucous were pale and no other physical abnormalities were diagnosed. Fecal flotation, complete blood count, bone marrow aspiration, cerebrospinal fluid (CSF) analysis, necropsy and histopathological examination were performed. Fecal flotation showed no helminth eggs or coccidial oocysts. On blood smears obtained from the earlap, >70 percent of erythrocytes were infected with piroplasms organisms and schizonts were obvious in smears of lymphocytes lymph node. Blood count revealed a lymphopenia, poikilocytosis, anisocytosis and non-regenerative anemia (packed cell volume; mean, 16%). Histopathological examination revealed Arthus reaction through the walls of cerebral blood vessels, which resulted in local necrosis of the brain. Analysis of CSF showed no abnormality in appearance or biochemical and cell counts. Although the calves were treated with a single intramuscular injection of buparvaquone and oxytetracycline once daily they did not respond to the treatment and died. In conclusion, the present cases showed a rare cerebral form of theileriosis by vertical transmission that confirmed by the presence of piroplasms on blood films and multinuclear schizonts on lymph node aspiration smears, gross and histopathological examinations and unsuccessful treatment in a newborn twin Holstein calves.

## Introduction

Tropical theileriosis (Mediterranean coast fever) is an endemic disease of cattle in Mediterranean basin and parts of Asia.^1^^,^^2^ A nervous form of the disease, known as cerebral theileriosis (turning disease), may develop in cattle.^3^^,^^4^ Cerebral theileriosis is manifested by ataxia, hypermetria, conscious proprioceptive deficits, circling, depression, head pressing, opisthotonos, nystagmus, blindness, hypersthenia, and convulsions. In this form of theileriosis, the animals become recumbent and develop severe opisthotonos, tonic-clonic seizures and coma.^4^^,^^5^ In rare cases the parasite may localize in the spinal cord.^2^^-^^4^ This paper examines the clinical and laboratory data from a rare case of newborn twin Holstein calves with cerebral theileriosis which result in local necrosis of the brain following Arthus reaction through the walls of cerebral blood vessels.^5^ There were no report or study similar to the present case from cattle in previous reports.

**Case presentation**

An 8-day-old newborn female twin Holstein calves with a history of weakness, anorexia, emaciation and convulsion were presented to Tabriz University Veterinary Teaching Hospital. The calves were referred from a semi-industrial dairy farm around Tabriz suburb. In clinical examinations, recumbency, depression, severe opisthotonos, nystagmus, pedaling, blindness, hypersthenia, pale buccal and vaginal mucous mucus membranes, sunken eyes, coughing, severe jaundice in mucus membranes, profuse salivation, lacrimation, dyspnea, diarrhea and dehydration were observed. The calves had been normal at the birth time and showed ataxia and blindness within five days after it. The calves were febrile (38.5 to 39 ˚C) and physical examination revealed heart rate of 124 beats per min, respiration rate of 68 breaths per min, extended heart sound to the thorax and flank, pale buccal and vaginal mucous with numerous petechial hemorrhages. Respiratory tract examination revealed dyspnea with moist rale (crackles) and occasionally apnea and palpation of the trachea caused coughing. Laboratory evaluation included a complete blood count (Hospitex Diagnostics, Florence, Italy), and fecal flotation test (with saturated sugar solution) for the detection of parasitic infections. In addition, blood smears on the glass slides were obtained from marginal veins of the earlap of both calves, fixed in methanol 10%, stained with Giemsa and examined for the presence of* Theileria* piroplasms. Urine samples were obtained from one of the calves and analyzed by tape urine analysis. Cerebrospinal fluid (CSF) samples were collected from lumbosacral cisterna by a 14 gauge needle. Although initial supportive treatment with a single intramuscular injection of buparvaquone (Razak Co., Karaj, Iran) and oxytetracycline (Razak Co., Karaj, Iran) once daily were performed the animals’ condition did not improve and ultimately, the calves died after two days. Necropsy was performed within 6 hr of death. Samples of the liver, spleen, kidney, intestine, lung and brain were collected and fixed in 10% buffered formalin for 24-72 hr. The samples were cut into 5 µm sections after embedding in paraffin. These sections were stained with hematoxylin and eosin and finally evaluated by a veterinary pathologist under the light microscope.

## Results

**Hematological and urological findings** **. **Complete blood count revealed hemoglobinemia, lymphopenia and severe non-regenerative anemia (packed cell volume, PCV 16%), (Table 1). Morphology of red blood cells (RBCs) exhibited polychromasia, poikilocytosis, anisocytosis, Howell-Jolly body and basophilic stippling. Nearly 70 percent of piroplasm organisms in erythrocytes were rod shaped. Samples of CSF were normal and did not have turbidity or other abnormalities. Urinalysis showed dark reddish brown urine in appearance, proteinuria (trace), bilirubinuria (2+) and hematuria (2+).

**Table 1:** Complete blood counts of the calves with cerebral theileriosis

**CBC Test**	**Calf-1**	**Calf-2**	**Normal ** *
Packed cell volume (%)	10.0	23.0	24.0- 46.0
Hemoglobin (g dl^-1^)	3.0	7.0	8.0- 15.0
Red blood cell (10^6^ µL^-1^)	1.1	2.7	5.0- 10.0
Mean corpuscular volume (fL)	90.9	85.1	40.0- 60.0
Mean corpuscular concentration (%) 30.0		30.4	30.0- 36.0
White blood cell (10^3 ^µl^-1^)	6500	6500	4000-12000
Neutrophil ( % )	47.0	11.0	1.0- 45.0
Lymphocyte ( % )	52.0	82.0	48.0- 75.0
Monocyte ( % )	1.0	7.0	2.0- 7.0

* All reference ranges were calculated based on data obtained from Jain NC Schalm’s Veterinary Hematology.^6^

**Macroscopic pathological and parasitological findings** **. **At necropsy, the carcasses were apparently cachectic, edematous, pale and relatively icteric. Free bloody fluids without fibrin and clot were found in abdomen and pericardium. Numerous petechia were evident on mucus membranes especially on palpebral conjunctiva, third eyelid and under the tongue. In one of the calves, prominent swelling was seen on superficial lymph nodes such as left prescapular, precrural, iliac and retropharyngeal (8 cm in diameter) lymph nodes. On transverse section of the lymph nodes, congestion, edema, red-brown cortex with numerous focal hemorrhage and dark red-brown medullary area were observed. Hepatomegaly with distended gall-bladder, yellowish brown color, rounded edges and sub-capsular focal hemorrhages in liver were clearly apparent. The kidneys were congested with cortical hemorrhages and moist on cut surfaces. On macroscopic examination of gastrointestinal tract, ulcerative abomasitis were observed. The ulcers were irregular in shape and various in size (2 to 10 mm in diameter) and appeared as punched-out craters surrounded by elevated rolled edges and involved most of the gastric mucosa. Gross examination of CNS revealed edema, congestion, petechial hemorrhages of the brain and meninges.

**Fig. 1:**
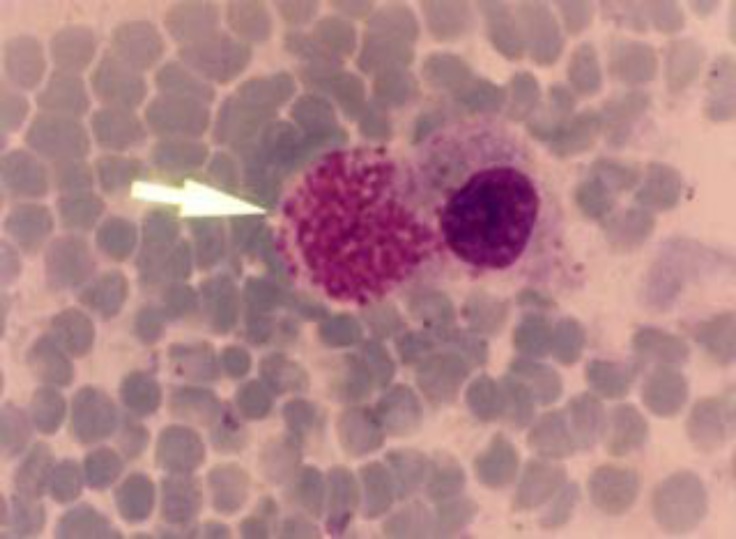
Presence of Koch's blue bodies in a contact smear of liver. The arrow indicates a lymphocyte containing numerous macroschizont (Giemsa and H & E, 1000×).

**Microscopic** **pathological** **findings** **. **On tissue section of the bone marrow, liver, kidney and heart Koch’s blue bodies were present (Macroschizont, Fig. 1). The histopathological examination on lymph node and spleen smears demonstrated severe infiltration of lymphocytes with hemorrhage and fibrinous exudates throughout the cortical areas of nodes. Lymphocytolysis was prominent in germinal centers and there was a general loss of small lymphocytes with those that remained large and blastic.

Also severe edema and numerous erythrophages, large macrophages that have phagocyted parasite-infected erythrocytes, were observed (Fig. 2a). The lesions indicated hemorrhagic lymphadenitis, splenitis, massive lymphocytolysis and blastoid transformation. The ulcers of the abomasums were associated with necrosis of epithelium, adjacent epithelial hyperplastic lesions, edema, arteritis, hyaline thrombi, infiltration of polynucleated cells and hyperplastic submucosal lymphoid follicles (Fig. 2b). The bone marrow was hyperplastic with the remaining cells consisting of large, blastic, parasitized lymphocytes and atypical erythroblasts. Accumulation of macrophages containing hemosiderin and phagocyted infected and non- infected erythrocytes were present with majority of them in spleen, lymph nodes and bone marrow. There were intravenous accumulations of lymphoblasts (with and without schizonts) in the brain, with venous thrombi and perivascular infiltration by lymphocytes. Microscopically, diffuse gliosis, perineuronal and perivascular hemorrhages and/or edema, status spongiosis, hyaline thrombi in capillaries and arterials, activation of endothelial cells and sequestration of parasitized cells in vessels were noticed in brain (Figs. 2c-f). Numerous thrombus formations and parasite-infected erythrocytes and lymphoblasts sequestered in cerebral blood vessels were also apparent on brain histopathological section (Fig. 2e). Arthus reaction was also present through the walls of cerebral blood vessels, which resulted in local necrosis of the brain.

**Fig. 2:**
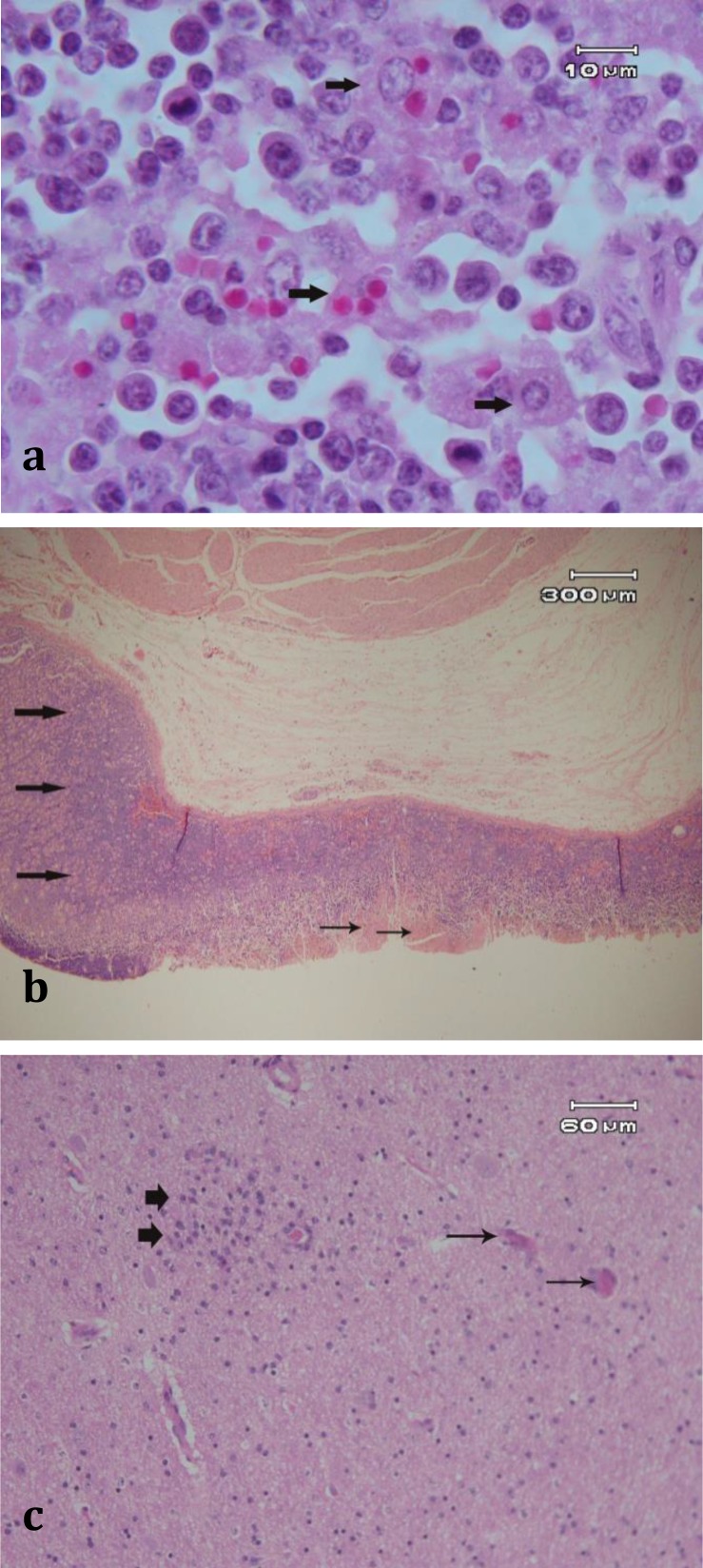
Photomicrographs of various tissue specimens from the calves died from severe theileriosis due to *T. annulata*.  **a.** Spleen, extravascular hemolysis with abundant large macrophages containing non-parasitize or parasite-infected erythrocytes (arrows), (H & E, 1000×); **b.** Ulcerative abomasitis, necrosis of the epithelium are present (thin arrows). Adjacent epithelium was hyperplastic (thick arrows), (H & E, 40×); **c.** Hyaline thrombi and gliosis in the brain, diffuse gliosis (thick arrows), hyaline thrombi occluded the capillaries and arterioles (thin arrows), (H & E, 200×); **d.** Brain, status spongiosis (thin arrow), activation of endothelial cells and sequestration of parasitized cells in vessels are present (thick arrow), (H & E, 200×); **e.** Hyaline thrombus in a brain capillary, hyaline thrombus occluded the capillary (thick arrow), perivascular edema and sequestration of parasitized and lysed erythrocyte contains two piroplasm organisms (thin arrow) are present (H & E, 1000×); **f.** Perivascular edema and status spongiosis around brain arteriole (long thick arrows) and activation of the endothelial cells (short thick arrow) with sequestration of parasite-infected erythrocyte in the lumen are present (thin arrow), (H & E, 1000×).

**Fig. 2 continued:**
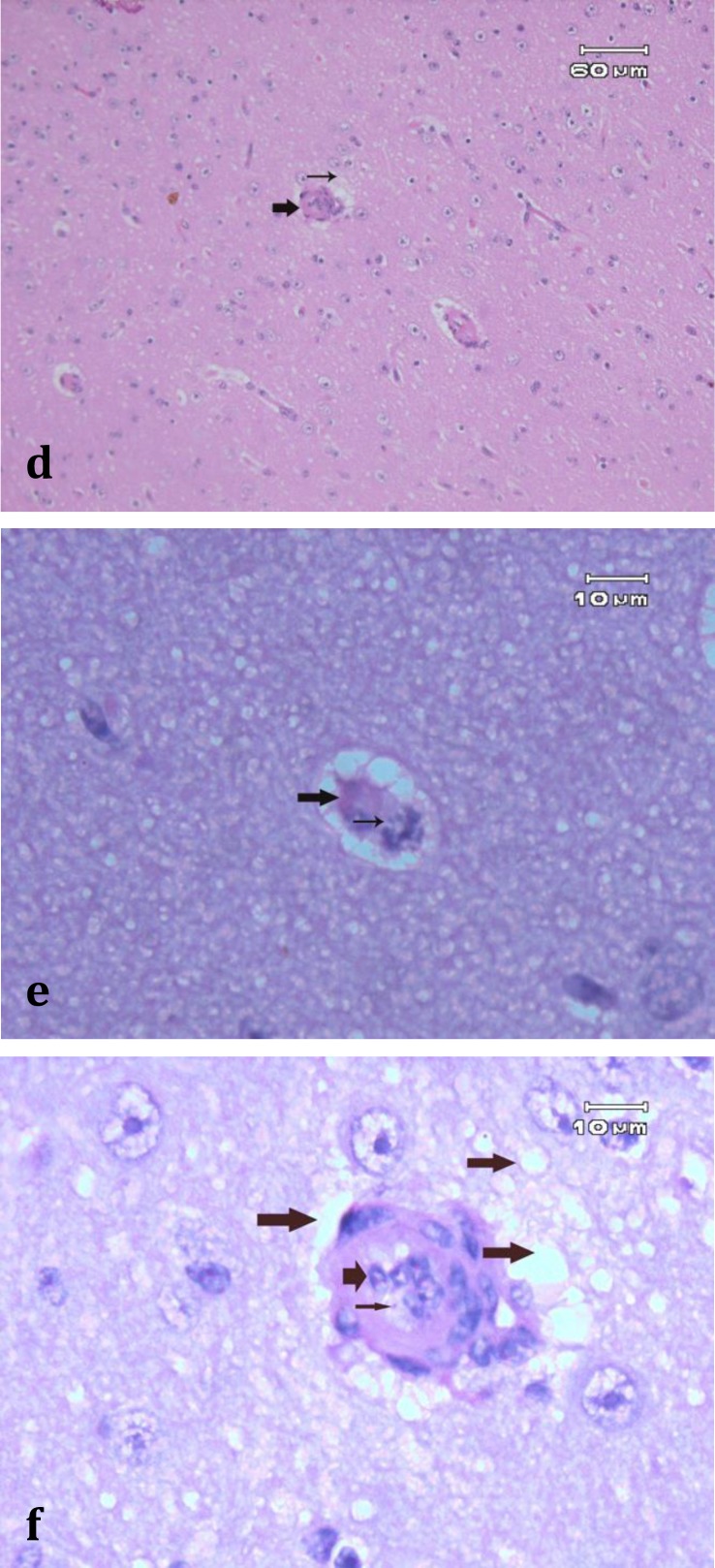
**d. **Brain, status spongiosis (thin arrow), activation of endothelial cells and sequestration of parasitized cells in vessels are present (thick arrow), (H & E, 200×); **e. **Hyaline thrombus in a brain capillary, hyaline thrombus occluded the capillary (thick arrow), perivascular edema and sequestration of parasitized and lysed erythrocyte contains two piroplasm organisms (thin arrow) are present (H & E, 1000×); **f. **Perivascular edema and status spongiosis around brain arteriole (long thick arrows) and activation of the endothelial cells (short thick arrow) with sequestration of parasite-infected erythrocyte in the lumen are present (thin arrow), (H & E, 1000×).

## Discussion

*Theileria* *annulata* causes tropical theileriosis which occurs in large parts of the world.^7^^-^^9^ The disease causes serious economic loss through bovine mortality and productivity loss.^10^ The differences between the prevalence of *T. annulata* among cattle of each province in Iran may be attributed to the different climate.^11^^,^^12^ The parasite has often been incriminated as the causal agent of cerebral syndrome, characterized by a number of nervous symptoms and, in particular, circling or turning movements which eventually result in death.^13^ The pathogenesis of the condition remains unclear. One possibility is that an auto-immune disorder induced by the parasite is responsible for intravascular agglutination of lymphocytes with subsequent embolism, thrombosis and infarction in the CNS.^3^ From our investigation it was concluded that *T. annulata* is the main cause of cerebral theileriosis in Iran. In South Africa, *T. taurotragi* is usually a benign parasite and it is not clear why it should sometimes cause cerebral theileriosis.^14^ Piroplasms of *T. annulata* are usually seen in mixed theilerial infections, but as they are easily distinguishable from those of other *Theileria* species occurring in Iran, they do not complicate the diagnosis of pathogenic *Theileria *spp.^11^ It is believed that this parasite occurs commonly within the distribution range of the genus *Amblyomma*, *Rhipicephalus* and *Hyalomma*,^11^ but as a result of its apparent insignificance very few reports exist to indicate its exact distribution. The clinical diagnosis of bovine cerebral theileriosis is difficult because of similarity in neurological signs to other neurological conditions. The diagnosis can be assessed by diagnostic methods such as hematology, lymph node aspirations, CSF collection, brain biopsy and electroencephalography (EEG). Hematology and lymph node aspirate analysis are completely unreliable. CSF samples from affected cattle shows increased protein content, a change which is associated with other neurological conditions. In these cases, the calves showed hemoglobinuria and severe decreases in PCV, hemoglobin and RBCs, indicating that it was suffering from a severe anemia. The anemia in these cases is more likely caused by intravascular hemolysis which is complicated with other disorders. The common clinical signs of the calves include severe opisthotonos, nystagmus, pedaling, blindness, hyperpnoea and hyper-sthenia. These findings have been reported in other studies by Saville and Dabak *et al*. in 2002 and 2004 in sporadic cases, respectively.^4^^,^^5^ Although it has been shown that buparvaquone is more effective in treatment the disease,^4^^,^^5^ we did not succeed to control the nervous form of the disease and the calves died after seven days of initial presentation. The damages occurred in theileriosis are related to both schizonts and piroplasms and the brain biopsy is reliable for diagnosis.^1^ In one study, schizonts were absent or rare in the lymph nodes, spleen, liver or lung and piroplasms were also rare in the blood, however, on brain impressions smears numerous schizonts were in the capillaries, either free or within lymphocytes.^13^ In another report, there were intravenous accumulations of lymphoblasts with and without schizont in the brain, with venous thrombi and perivascular infiltration by lymphocytes.^15^

The nervous signs occurred in the present cases were due to Arthus reaction through the walls of cerebral blood vessels that resulted in vasculitis and lymphocytic inflammation of the brain. These findings are in agreement with other studies in cerebral forms of theileriosis.^3^ In these cases, edema, petechial hemorrhages and congestion on the surface of the brain and meninges were reported as postmortem features of the cerebral theileriosis. Another forms such as thrombosis of the meningeal vessels hemorrhage in the cerebral ventricles and infarctions of the splenic vessels have been reported as postmortem features in other reports.^3^^,^^4^^,^^15^ The polymerase chain reaction (PCR) assay is the method used to diagnose theileriosis infection and is considered the “gold standard”.^6^^,^^14^ In one study, the PCR diagnostic tests were found to be more accurate and sensitive than both microscopic assays.^14^ Large variation in seroprevalence of *T. annulata* using other serological tests was found due to certain epidemiological factors, especially geographical distribution of biological vectors, the average age, lifestyle and clinical status of the cattle.^4^^,^^16^ Theileriosis can also be transmitted vertically, which in this place abortion of calves or the birth of affected calves with the disease will happen.

In conclusion, the diagnosis of the cerebral theileriosis with a congenital occurrence was confirmed by evaluation of the calves age, clinical signs, and blood examination as well as gross and histopathological examination. Demonstration of piroplasms and multinuclear schizonts in blood, spleen, liver or brain, thrombosis, infarction of nervous tissue as well as lymphocytic meningomyelitis and existence of local necrosis following Arthus reaction through the walls of cerebral blood vessels were the most remarkable findings. The present cases indicated that *T. annulata *was a cause of cerebral theileriosis, however, further studies using PCR need to determine the types of *Theileria* responsible for the cerebral form of the disease.
